# Accelerating the development of bioelectronic medicine: overview and impact of the NIH Stimulating Peripheral Activity to Relieve Conditions (SPARC) initiative

**DOI:** 10.1186/s42234-026-00212-0

**Published:** 2026-08-19

**Authors:** Katelynn A. Milora, Rebecca M. Black, Andrew C. Weitz, Michael B. Wolfson, Jessica Falcone, Eric Hudak, Brooks Gross, Nicholas Langhals, Sahana N. Kukke, Daniel Anthony Casco, Kristina Faulk, Julia Berzhanskaya, Wen G. Chen, Emrin Horgusluoglu, Felicia Qashu

**Affiliations:** 1 Office of Strategic Coordination (OSC), Office of the Director (OD), National Institutes of Health (NIH), Rockville Pike, Bethesda, MD USA; 2 National Institute of Biomedical Imaging and Bioengineering (NIBIB), National Institutes of Health (NIH), Rockville Pike, Bethesda, MD USA; 3 National Institute of Neurological Disorders and Stroke (NINDS), National Institutes of Health (NIH), Rockville Pike, Bethesda, MD USA; 4 National Heart, Lung, and Blood Institute (NHLBI), National Institutes of Health (NIH), Rockville Pike, Bethesda, MD USA; 5 National Center for Complementary and Integrative Health (NCCIH), National Institutes of Health (NIH), 6707 Democracy Blvd, Bethesda, MD USA

**Keywords:** SPARC, Vagus Nerve, Neuromodulation, Consortium, Autonomic Nervous System (ANS), Peripheral Nervous System (PNS)

## Abstract

The National Institutes of Health (NIH) Common Fund supports bold scientific programs that catalyze discovery across all biomedical and behavioral research to address high priority challenges for the NIH as a whole and make a broader impact in the scientific community. In 2015, the NIH Common Fund established the Stimulating Peripheral Activity to Relieve Conditions (SPARC) program. This program’s overall goal was to accelerate the development of neuromodulation, a field of study that uses bioelectronic devices to deliver electrical current to nervous system tissues to produce therapeutic effects in patients. Because the nervous system impacts all bodily functions, neuromodulation therapies have the potential to treat a variety of health conditions by regulating nerves that innervate affected bodily tissues and organs, with high specificity and few side effects. The SPARC program aimed to capitalize on recent advances in technology to deliver detailed, integrated functional and anatomical neural circuit maps for organs and to provide the necessary scientific foundation for clinical translation of more effective and advanced neuromodulation devices and stimulation protocols. Here we outline the SPARC program’s contribution to the field of neuromodulation over the past ten years, laying a comprehensive foundation of knowledge to inspire future research and accelerate translation. We discuss the reasoning behind the formation of the program, its structure, and outcomes.

## Introduction

There is a growing need for new treatment options for many debilitating diseases and chronic conditions. Pharmacological treatment options are often limited or ineffective for inflammatory and autoimmune diseases, obesity, diabetes, neurodegenerative and neuromuscular disorders, and gastrointestinal and genitourinary diseases. Nervous system modulation is currently being used in hundreds of ongoing clinical trials and offers a promising approach to treating such diseases where existing therapeutic options fall short (Pavlov et al. 2019; Pavlov and Tracey 2019; Fernandez 2018; Kovatchev et al. 2020; Olofsson and Tracey 2017; Bouton et al. 2016).

Neuromodulation is the use of advanced medical device technology to enhance or suppress the activity of the nervous system for the treatment of disease (International Neuromodulation Society n.d.). Neuromodulation as a field of study, uses bioelectronic devices to deliver electrical current to nerves and neurons to produce therapeutic effects in patients (Birmingham et al. 2014). Neuromodulation technologies may be implanted within the body or used externally to stimulate electrically active tissues, like the brain, spinal cord, peripheral nerves, heart, and other muscles. Because the autonomic nervous system (ANS) innervates all tissues of the body, neuromodulation has the potential to affect numerous organ systems and therefore treat a variety of diseases (Birmingham et al. 2014). Unlike with pharmacological treatments, bioelectronic devices may be used to selectively affect tissues local to the implant or at a distal site, when leveraging electrical conduits in the body, such as the vagus nerve (Peeples 2019; Mylavarapu 2023).

This emerging field has the potential for unique advantages in treating conditions where traditional pharmaceutical approaches produce a high level of side effects or are ineffective (Pikov 2023). Use of bioelectronic medicine in clinical practice can greatly benefit patients; however, these methods have faced unique challenges (Birmingham et al. 2014). A history of study failures, including the failure of transneuronix devices to consistently influence weight loss during clinical trials (Lebovitz 2015), resulted in limited investment and innovation (Birmingham et al. 2014). Lengthy clinical studies, questionable relevance of animal models, and challenges with national reimbursement coverage further decreased enthusiasm for investment. Technical limitations of studies were prevalent, such as imprecise targeting or lack of quantitative endpoints or biomarkers, which was driven by incomplete knowledge of mechanisms of therapeutic action. Substantial investments were needed to understand how the ANS interacted with organ systems to assess relevant therapeutic targets. Additionally, the field was in need of investment in new tools, technologies, and data resources that would enable researchers to easily leverage new findings to advance the field of neuromodulation.

## Conception of the program

In 2013, representatives from National Institutes of Health (NIH), the Defense Advanced Research Projects Agency (DARPA), and GlaxoSmithKline organized a “Bioelectronic Medicines Summit” to bring together experts from academia, industry, and the federal government to discuss the field of neuromodulation. The feedback from the meeting and pre-meeting webinars resulted in a research roadmap for bioelectronic medicines that envisioned a firm research foundation for therapies that modulate the electrical signaling patterns in the ANS. Critical priority areas included 1) the need for biological maps of structure and function, 2) advancements in interface technology, 3) early establishment of therapeutic feasibility, and 4) collaborative data mining and standards enabled by an “emerging community of bioelectricians.” (Birmingham et al. 2014) Informed by this roadmap and additional resources and discussions, the NIH formed a Working Group that developed a concept proposal for a new Common Fund program called Stimulating Peripheral Activity to Relieve Conditions, or SPARC.

The proposed concept and goals for the SPARC program were cleared by the NIH Council of Councils in June 2014. The SPARC program was consequently initiated by the NIH Office of the Director (OD) in partnership with the National Center for Advancing Translational Sciences (NCATS), the National Institute of Biomedical Imaging and Bioengineering (NIBIB), the National Institute of Diabetes and Digestive and Kidney Diseases (NIDDK), and the National Institute of Neurological Disorders and Stroke (NINDS).

**Fig. 1:**
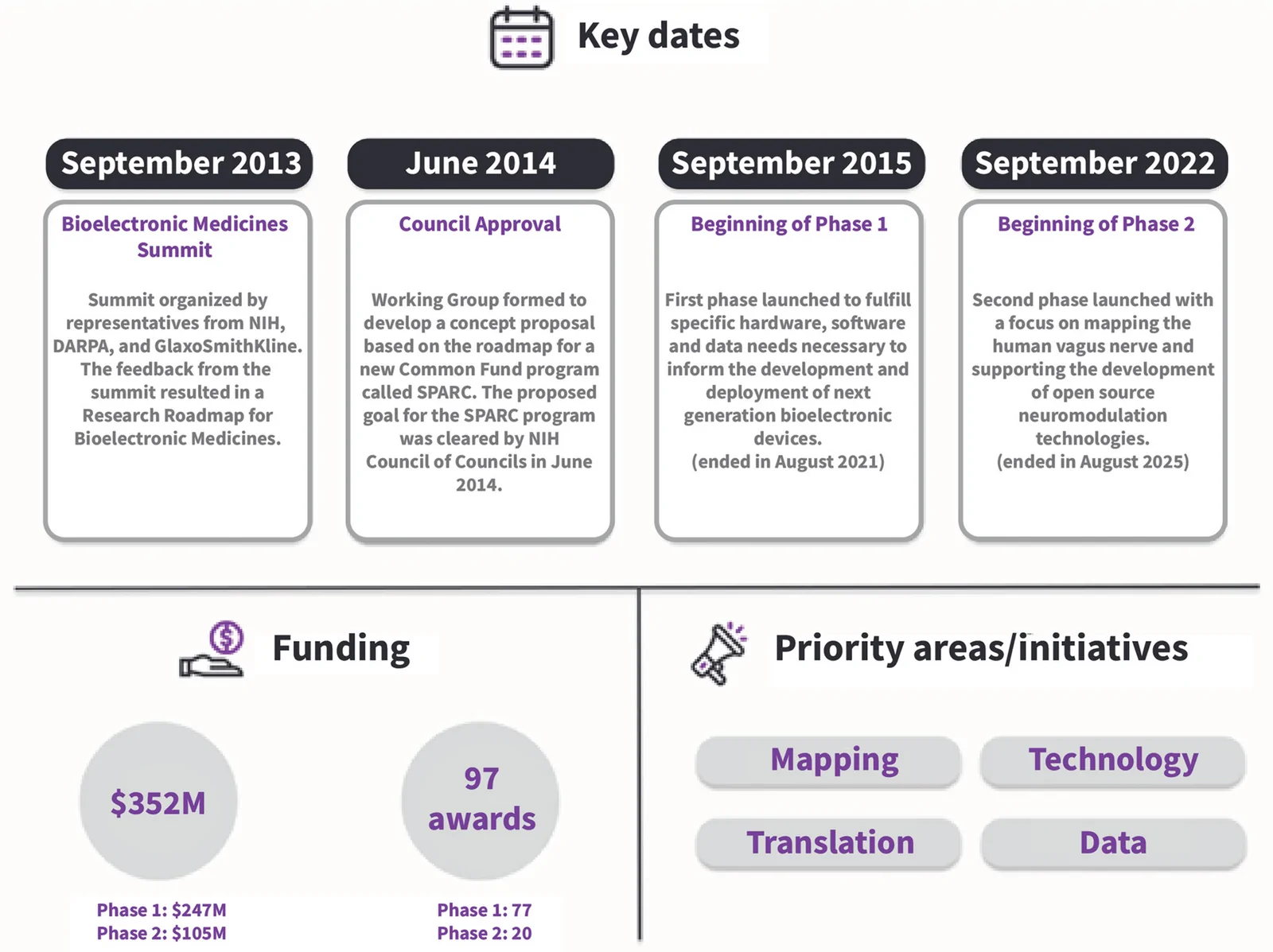
Overview of the SPARC program through key dates, funding, and priority initiatives

The goal of the SPARC program was to accelerate the development of therapeutic devices that modulate electrical activity in peripheral nerves to improve organ function. To achieve this overall goal, SPARC was launched in two consecutive phases (Fig. 1).

## Design

### Phase 1

By September 2015, SPARC launched its first phase, which spanned seven years (fiscal years (FY) 2015–2021). Four major interdependent initiatives covering four priority areas were established (Table 1):

1. Mapping. The SPARC mapping initiative aimed to support the creation of new anatomical and functional connectivity datasets. These would facilitate hypothesis generation and testing in areas such as the coursing and branching of peripheral nerves, the distribution of axon terminals, nerve-organ synapses, the cross-sectional organization of nerves, the functional effects related to an internal organ mediated by firing patterns, and the relevance of particular animal models to human systems (Leung 2021, 2023, 2020; Settell 2020; Ludwig 2022a, b; Wiedmann et al. 2021; Keast 2020; Bertrand et al. 2020).
2. Technology. The SPARC technology initiative fostered the development of cutting-edge tools and technologies for studying and interfacing with the ANS and internal organs, which in turn supported other SPARC initiatives (Damaser 2022a, b, 2023a, b; McAdams 2018a, b; Wang et al. 2021; Gregersen 2022; Wang 2023a, b; Musselman et al. 2021; Marshall et al. 2023a; Marshall et al. 2023c).
3. Translation. The SPARC translational initiative explored new indications for existing devices and funded clinical studies to gain greater mechanistic understanding of targeted neuromodulation therapeutics (Kusayama 2021; Jiang 2018; Ouyang 2022; Zhang et al. 2019).
4. Data. The SPARC Data initiative supported the development of tools and resources for making all SPARC-funded data, models, and other research outcomes FAIR (Findable, Accessible, Interoperable, Reusable) (Wilkinson 2016). This included the development of the SPARC Portal (https://sparc.science) (SPARC Portal n.d.), where all SPARC-supported outcomes are openly available to the scientific community (Osanlouy 2021; oSPARC n.d.; oSPARC Documentation n.d.; Osparc-Simcore: Osparc-Simcore Simulation Framework n.d.).

**Table 1:** Structure of the SPARC program around priority areas/initiatives during Phase 1 and Phase 2

Priority initiatives	SPARC Phase 1 (FY 2015–2021)	SPARC Phase 2 (FY 2022–2024)
Mapping	Mapping of five organ systems (heart, lungs, stomach, colon, bladder), understudied organs (spleen, pancreas, esophagus, kidney, liver, adipose tissue, bone, bone marrow), and pain circuits	Mapping of the human vagus nerve
Technology	Tools and computational models to improve optimization and targeting specificity of stimulation, sensors to detect changes in autonomic nerves or internal organs	Open source neurotechnologies
Translation	Explorations of new indications for existing devices, clinical trials to gain a greater mechanistic understanding of the targeted neuromodulation therapeutic	Advancement of neuromodulation technologies that can target multiple autonomic functions
Data	SPARC Data and Resource Center (DRC), data standards, anatomical and functional connectivity maps, connectivity knowledgebase, computational models, cloud simulation platform, SPARC Portal (https://sparc.science)	Improved data visualization, interaction, and modeling tools, variability quantification, in silico safety & efficacy assessment, automations for maps, sustainability of resources and capabilities on the SPARC Portal and associated infrastructure/tools

A total of $247 million (M) was distributed during Phase 1 through 77 awards across these four initiatives.

### Phase 2

Towards the end of SPARC Phase 1 in 2020, the NIH SPARC Working Group assessed the impact of SPARC and recognized wide-ranging progress being made across the four priority areas. Use of novel techniques, tools, and large animal/human tissue in mapping studies were furthering our understanding of the ANS and internal organ connectivity. New therapeutic strategies were emerging with proof-of-concept studies exploring new applications and state-of-the-art modalities of neurostimulation. Data, maps, and models were being shared in a FAIR way through the SPARC Portal to support a neuromodulation knowledge base and artificial intelligence (AI) driven device development. To build on the progress and momentum of Phase 1 (Table 2), the NIH SPARC Working Group sought strategic input from potential partners to identify additional high-impact collaborative goals (through a request for information and a stakeholder meeting with leading academic, industry, and government partners) and developed additional initiatives for the SPARC program. After receiving Council of Councils clearance in January 2021, SPARC launched a three-year Phase 2 in FY 2022. The goal of Phase 2 was to support further progress in the four priority initiatives of SPARC Phase 1 but with a strategic focus on the vagus nerve (Table 1). The vagus nerve was selected because it innervates many internal organs and is an established therapeutic target; yet vagus nerve stimulation (VNS) still has breakthrough potential due to the rudimentary understanding of current treatments.

**Table 2:** Notable early outcomes across the SPARC priority areas

Team location	Outcome
*Mapping*	
University of Central Florida and Thomas Jefferson University	Developed a comprehensive 3D atlas of the rat intrinsic cardiac nervous system (ICN) at the single-cell resolution to characterize the distribution patterns of intrinsic cardiac neurons between individuals and across sexes (Leung 2021, 2023, 2020).
University of Wisconsin-Madison and Duke University	Combined microdissection, histology, and immunohistochemistry to provide data on key features across the cervical vagus nerve in a swine model and then compared it to other animal models (mouse, rat, dog, non-human primate) and humans (Settell 2020; Ludwig 2022a, b).
University of Melbourne	Used immunohistochemical immediate-early gene (IEG) markers to map neuronal activity in lumbosacral spinal cord after stimulating intense micturition in awake, unrestrained female and male rats, and showed sex difference (Wiedmann et al. 2021; Keast 2020; Bertrand et al. 2020).
*Technology*	
Case Western Reserve University	Developed bladder and bowel monitors to wirelessly transmit organ function and demonstrated effectiveness in conscious ambulating animals for potential closed loop or triggered stimulation (Damaser 2023a, b, 2022a, b; McAdams 2018a).
California Medical Innovations Institute	Developed, clinically validated, and received FDA regulatory 510(k) clearance for Fecobionics, a simulated feces that provides an objective, safe, and low-cost assessment of defecatory function by measuring a variety of parameters during defecation of the device including pressures, bending, and shape changes which can be used for improved diagnosis of anorectal disorders including constipation and fecal incontinence (Wang et al. 2021; Gregersen 2022; Wang 2023a, b).
Duke University	Created ASCENT (Automated Simulations to Characterize Electrical Nerve Thresholds), a pipeline for sample-specific computational modeling of electrical stimulation of peripheral nerves which is used to virtually design experimental and clinical neuromodulation interventions (Musselman et al. 2021; Marshall et al. 2023a, b; Marshall et al.2023c; Musselman 2023a, b, c, d; Davis 2023).
*Translation*	
Indiana University	Used a canine model of atrial fibrillation (a type of arrhythmia) to compare the effectiveness of VNS and sympathetic nerve stimulation in slowing down the ventricular rate and determined that high-intensity sympathetic nerve stimulation was more beneficial for treating atrial fibrillation (Kusayama 2021; Jiang 2018).
University of Michigan	Used a feline model of overactive bladder to compare the effectiveness of open-loop sacral nerve stimulation (SNS) and closed-loop SNS, where bladder sensory feedback was recorded from dorsal root ganglia, and demonstrated superior performance of closed-loop SNS (Ouyang 2022).
Johns Hopkins University	Applied spinal cord stimulation at the thoracic level in a canine model of gastroparesis and observed normalization of gastric motility along with suppression of sympathetic overactivity (Zhang et al. 2019).
University of Pittsburgh	Developed a multichannel implantable device for sacral-pudendal neuromodulation in patients with bladder, bowel, and sexual disorders and, through an IDE-enabling study, generated the safety and efficacy data needed to advance the therapy toward regulatory approval and clinical use (Neuromod Prize n.d.).
Juniper Biomedical	Developed a highly precise, micro-implantable neuromodulation device to treat stress urinary incontinence, overactive bladder, and fecal incontinence and demonstrated its readiness to be considered for regulatory approval and clinical use (Neuromod Prize n.d.).
*Data*	
The SPARC Data Resource Center (DRC), IT’IS Foundation, University of Auckland, University of California San Diego, University of Pennsylvania	Developed the SPARC Portal, which was launched at the International Society of Autonomic Neuroscience (ISAN) meeting in July 2019 and has been actively expanded since (SPARC Portal n.d.). In a move towards sustainability for the SPARC Portal, DRC PIs expanded focus from the ANS to the PNS and developed resources and materials for investigators to submit their data to SPARC in preparation for the NIH data sharing mandate in January 2023. The DRC now supports non-SPARC investigators and continues to develop new resources and methods to support these new members of the SPARC community
University of Pennsylvania	The datasets on the SPARC Portal are hosted through the Pennsieve platform, which was initially developed at Blackfynn Inc. In 2021, the platform was made open-source and further development and maintenance of the platform and associated projects were transferred to the University of Pennsylvania
IT’IS Foundation	The computational models on the SPARC Portal are hosted through o^2^S^2^PARC, an open, online-accessible, cloud-based platform that supports collaborative, reproducible, sustainable, and FAIR elaboration, sharing, publication, execution, and deployment of simulations, data analysis, expert workflows, and applications (Osparc-Simcore: Osparc-Simcore Simulation Framework n.d.). For example, the interoperability, reusability, and collaborative modeling capabilities of o^2^S^2^PARC were leveraged to combine AI-based creation of personalized human spinal cord models from medical image data, electromagnetic simulations, neural dynamics modeling, and efficient optimization techniques to design and guide research on a bioelectronic implant for bladder control

A total budget of $105M was dedicated across 24 awards. In addition to the program focus on functional connectivity of the human vagus nerve, Phase 2 also aimed to build a new ecosystem of open-specification neuromodulation device components. This venture aimed to accelerate neuromodulation studies and reduce regulatory burdens on researchers by creating open-source hardware, software, firmware, and other modules needed to build neuromodulation systems. Finally, Phase 2 of SPARC included the Neuromod Prize (Neuromod Prize n.d.), a $9.8 million, multi-phase competition to develop breakthrough peripheral nerve stimulation technologies to independently regulate two or more desired autonomic functions without unintended effects on non-target organs. Eight winners were awarded the first phase prize and continued through to second phase, where winners conducted proof-of-concept studies. Four winners advanced to the third phase, where two final winners were selected and announced in fall 2025 based on their technology’s high safety and efficacy as required for an Investigational Device Exemption (IDE) and/or a clinical trial and the likelihood of maturing along the commercial pathway for clinical use.

## SPARC program metrics

### Overall performance

**Fig. 2:**
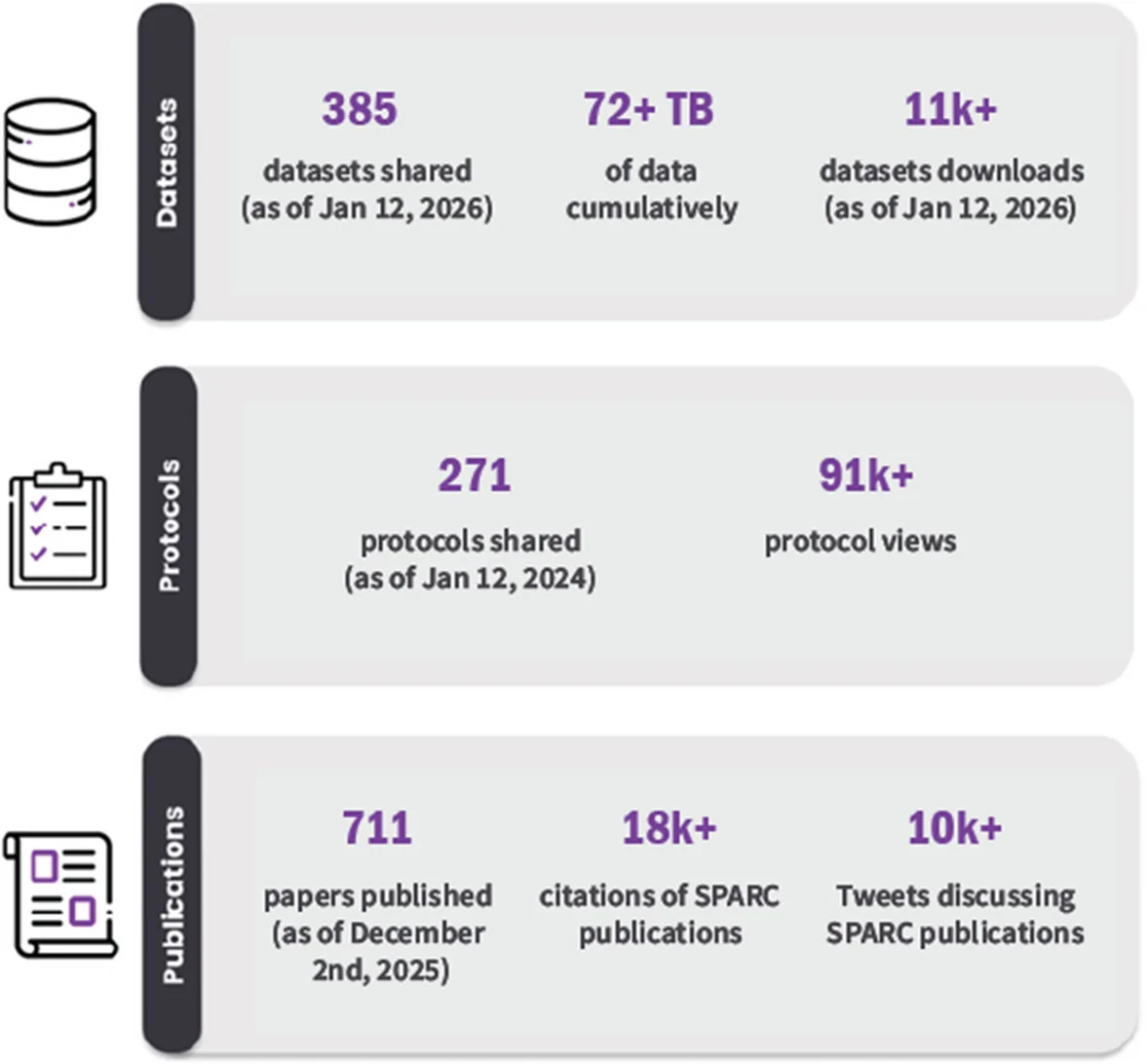
Key metrics demonstrating the overall performance of the SPARC program’s data and knowledge dissemination

**Fig. 3:**
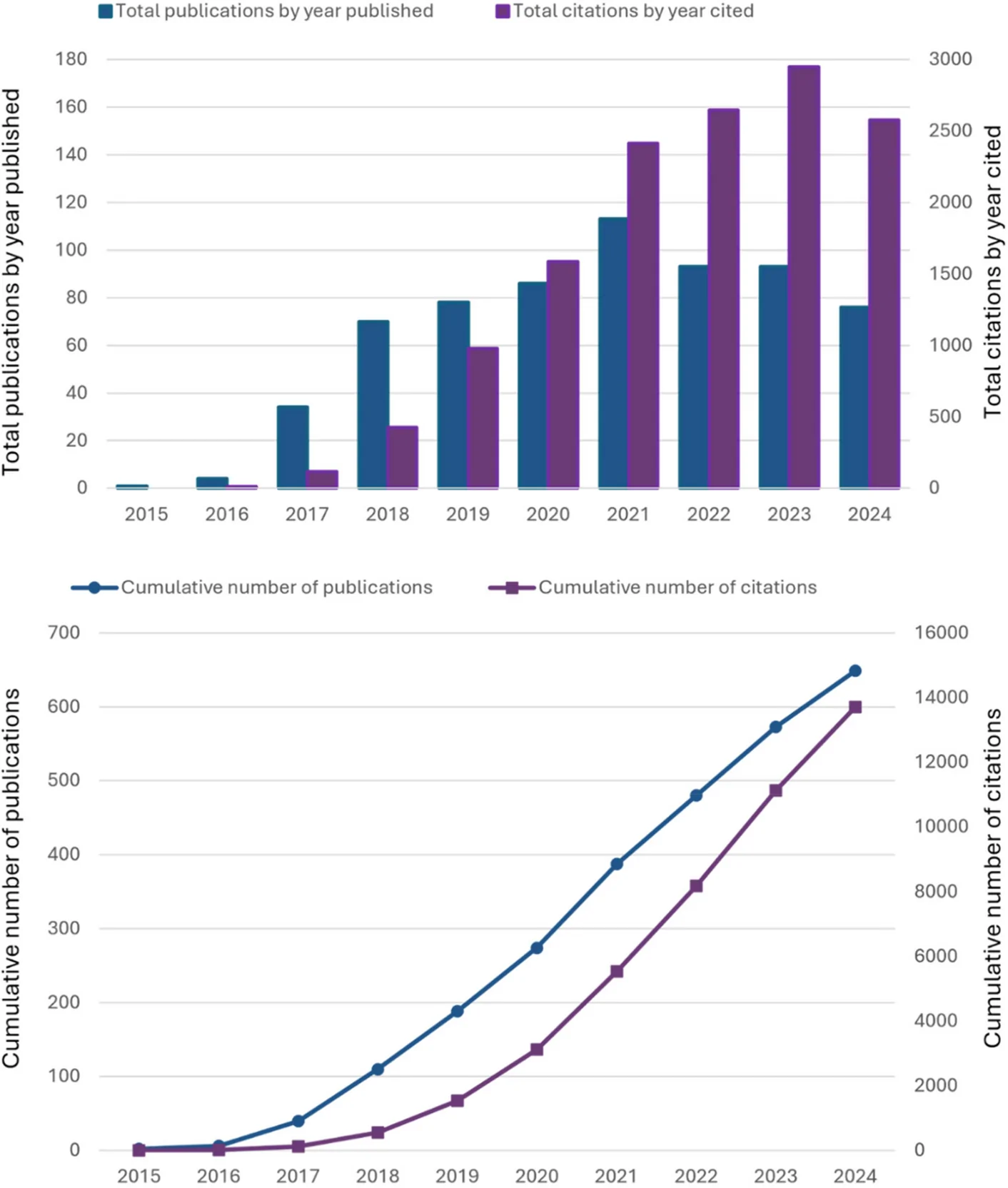
SPARC publications and their impact

Since the SPARC program began in 2015, it has produced hundreds of datasets, protocols, and publications, with at least 711 research articles citing SPARC Phase 1 grants published as of December 2, 2025 (Fig. 2). The number of SPARC-funded publications has grown steadily each year, and this body of work has been cumulatively cited over 18,091 times in the literature with a weighted relative citation ratio (RCR) (Hutchins 2019) of over 100 every year since 2017 (Table 3). (Fig. 3). As of January 23, 2025, 522 articles within the SPARC portfolio had Altmetric’s “attention scores”, and 250 articles (38% of the entire portfolio) have above average attention scores normalized to the time period and journal the articles were published in, and 37 articles (5.6% of the entire portfolio) are in the top 5% attention score. 14,397 posts on the social media website X have discussed SPARC publications, and SPARC research articles have been mentioned in 1,575 news articles. Details about calculating these metrics are provided in the Methods section.

**Table 3:** Relative Citation Ratio (RCR) of SPARC publications per year published

**Year**	2015	2016	2017	2018	2019	2020	2021	2022	2023	*2024	*2025
Number of Publications	1	4	38	70	79	86	118	97	104	74	6
RCR	0.6	9.12	140.2	148.4	169.6	144.1	167.3	140.2	183.6		

^*^It is best practice to allow at least two years between publication and citation analysis to allow uptake in the literature, so the RCR for 2024 and 2025 are not included

### Cross-disciplinary collaborations

The SPARC program was developed with the infrastructure to facilitate partnerships across various initiatives, with the goal of breaking silos and enabling novel discoveries in the bioelectronic medicine field through collaboration across disciplines. Throughout the duration of the SPARC program, SPARC researchers were able to work across teams to ensure developments were not siloed. This was facilitated by the design of the program through networking and collaboration opportunities arising from in-person Principal Investigator meetings and by ad hoc connections coordinated by NIH program staff. For example, Mapping teams tested early Technology prototypes and gave end-user feedback to Technology teams. Technology teams worked with Mapping teams to understand what may be needed to improve capabilities and make cutting edge advancements in their mapping research. Modeling supported technology safety assessment and efficacy optimization (e.g., stimulation and sensing). Translation teams leveraged devices and sensors from technology groups in large animal and human studies. Modeling teams worked with the Mapping teams to make sure that the anatomical and functional connectivity data generated would be most useful for modeling peripheral nerve circuits or stimulation. All teams collaborated with the SPARC Data and Resource Center to facilitate sharing large datasets across teams, even before final datasets were ready to share with the public.

To quantify these collaborations, SPARC publications from Phase 1 were analyzed using Cytoscape (Cytoscape n.d.). From the beginning of the program through 2019, SPARC researchers that co-authored more than one paper with another SPARC author averaged approximately 6 co-authorships with other SPARC PIs. In the second half of Phase 1 (2019–2021, and 2022 through no cost extensions of Phase 1 awards), the co-authorship networks grew much larger and less dense, nearly doubling the size of the diameter of the largest sub-network. Overall, SPARC authors were on average connected to 9 other authors, and the overall co-authorship space was much less centralized and dense (Table 4, also see full Cytoscape network file in the dataset related to this work described in the Data Availability section). Three of the largest connected networks from the first half of Phase 1 became strongly connected in the second half. This included some of the most central researchers involved in projects across SPARC mapping and translation initiatives. It is promising to see the potential for increased information flow between researchers. Details about the use of Cytoscape are provided in the Methods section.

**Table 4:** Cytoscape network summary statistics of SPARC Phase 1 co-authorship networks

Year	2015–2019	2020–2023
# Nodes	232	541
# Edges	585	1916
Avg. # of neighbors	5.897	9.177
Network diameter	4	7
Characteristic path length	2.121	3.479
Network density	0.155	0.053
Network centralization	0.696	0.249

Beyond collaborating across disciplines with the SPARC program, the SPARC Data and Resource Center also engaged with the Common Fund Data Ecosystem (CFDE, https://commonfund.nih.gov/dataecosystem)(CFDE n.d.), which links users to datasets across Common Fund programs. By ensuring that SPARC metadata and processed data were searchable through the CFDE Data Portal (https://data.cfde.cloud/), the reach and interoperability of SPARC data with other biomedical datasets was effectively expanded.

### Increased support across NIH institutes

**Fig. 4:**
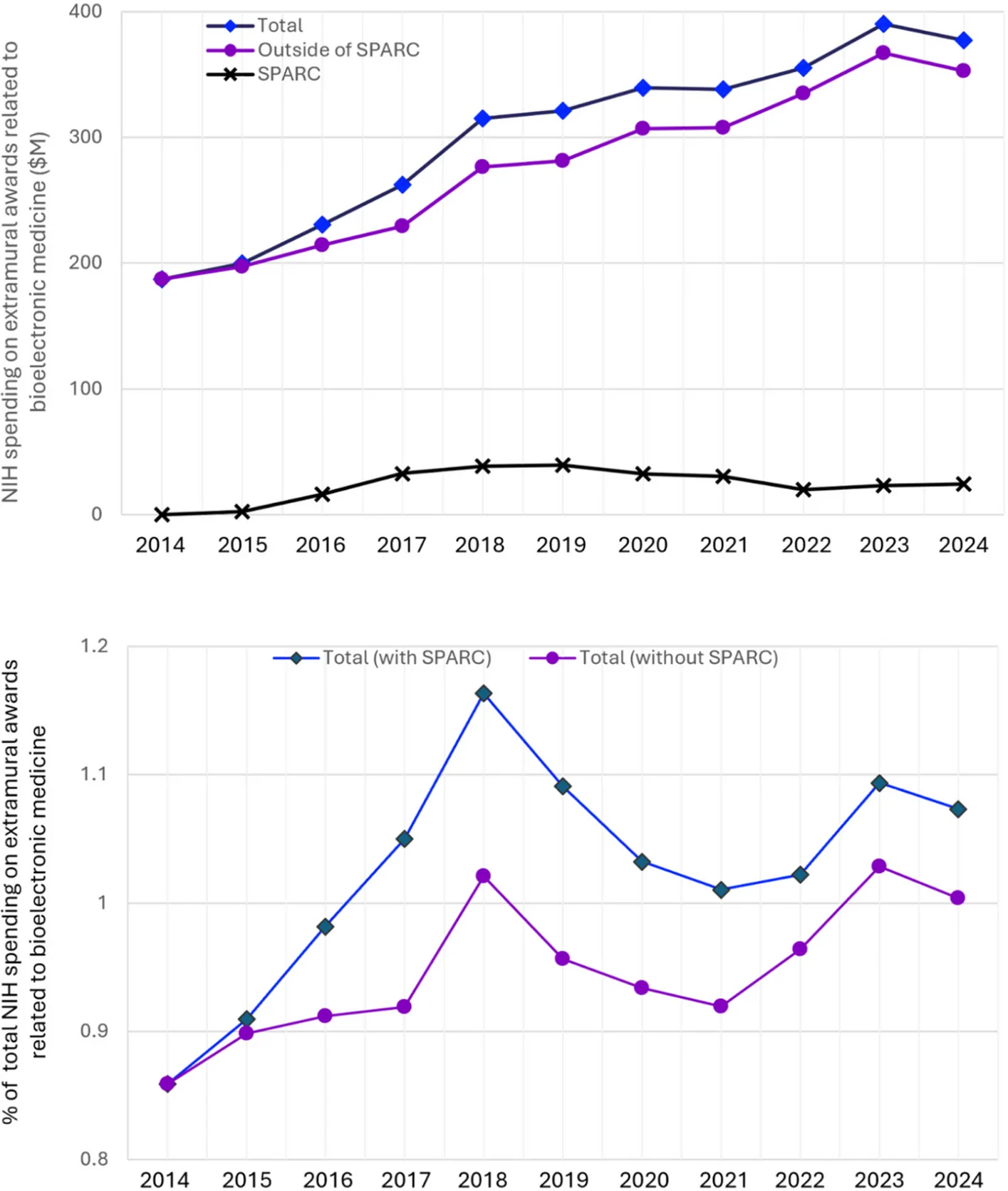
Evolution of NIH spending on extramural awards related to bioelectronic medicine since the inception of the SPARC program

**Fig. 5:**
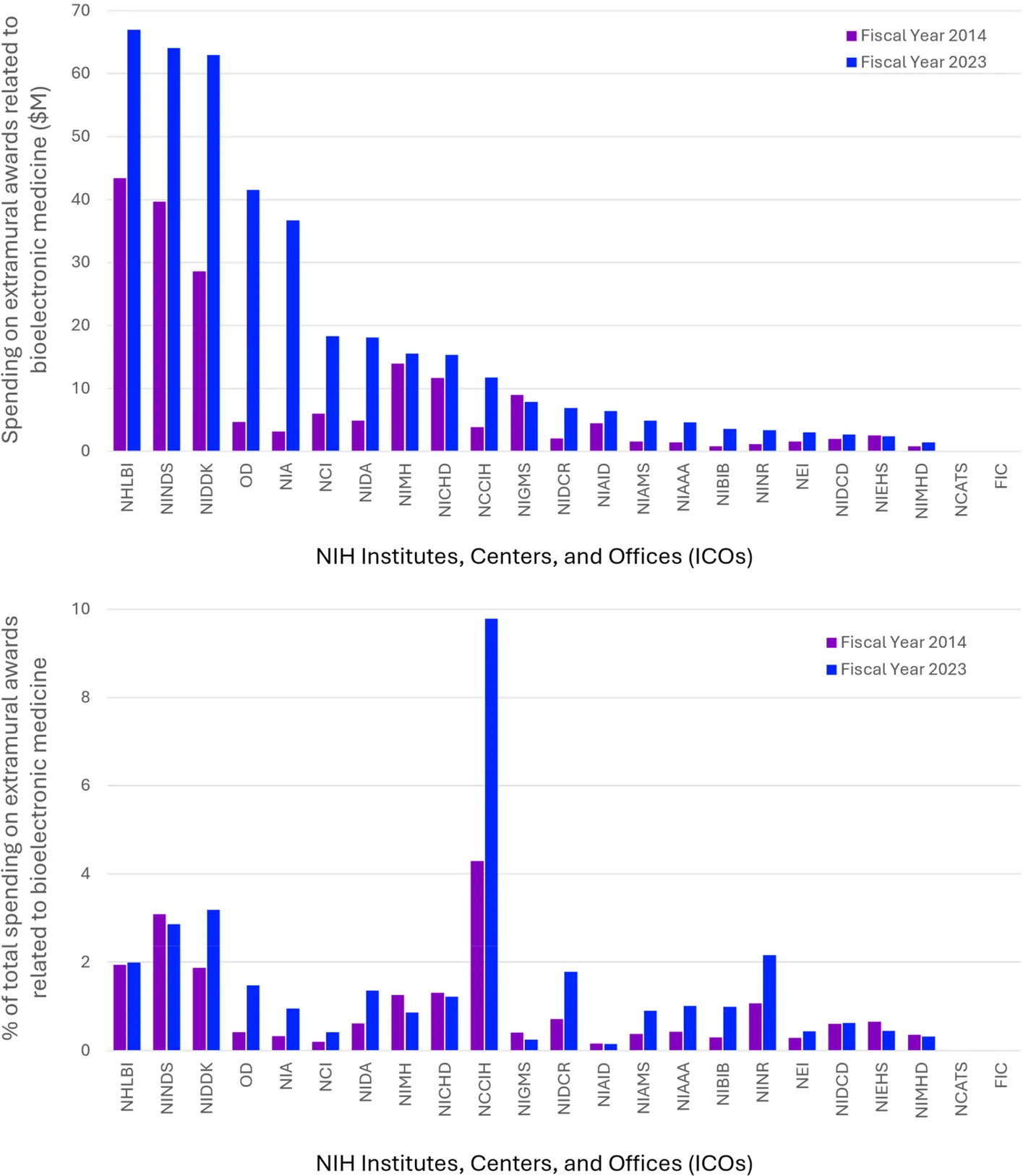
Evolution of NIH spending on extramural awards related to bioelectronic medicine per ICO between FY 2014 (before inception of the SPARC Program) and FY 2023

The specific effects of the SPARC program on the broader scope of neuromodulation research funded by NIH are difficult to separate from bioelectronic medicine research outside of SPARC. However, the direction of the SPARC program and the impact of its research outputs affect the neuromodulation research landscape and are in turn informed by external trends. Since the inception of the SPARC program, neuromodulation research across the NIH landscape has grown and expanded across nearly all NIH Institutes, Centers, and Offices (ICOs). Between FY 2014 and 2023, NIH research spending on extramural awards increased by 64% overall, while spending related to bioelectronic medicine specifically increased by 96% (excluding SPARC awards) and113% (including SPARC awards). A year-wise analysis shows that spending related to bioelectronic medicine has increased every year between FY 2014 and FY 2023, except for 2021 (Fig. 4). However, from 2019–2021, bioelectronic medicine awards declined as a percentage of all overall spending, while still remaining above FY 2014 levels. Nearly all NIH ICOs with these types of awards in bioelectronic medicine in FY 2014 increased their spending in this area by 2023 (Fig. 5). The National Heart, Lung, and Blood Institute (NHLBI), NINDS, NIDDK, OD (particularly the Common Fund), and National Institute on Aging (NIA) funded the most awards in this area in 2023, with notable growth in total awards in this area for NIA and NIDDK. As a fraction of their overall spending on these types of awards, the National Center for Complementary and Integrative Health (NCCIH), NIDDK, and the National Institute of Nursing Research (NINR) have some of the highest representation of bioelectronic medicine research in their portfolio, with a dramatic spike in the number of NCCIH’s awards in this area in the last decade (Fig. 5). Overall, changes within individual ICOs since FY 2014 show shifts in how some areas of NIH focused on pain treatment have prioritized research in this area in the last nine years. Details about obtaining the NIH spending data are provided in the Methods section.

### Continued investment in neuromodulation technologies at NIH and beyond

The SPARC program fits in a continuum of NIH investments in neuromodulation technologies. SPARC, which focuses on the peripheral and autonomic nervous systems, ran in parallel to the NIH Brain Research through Advancing Innovative Neurotechnologies (BRAIN) Initiative, which focuses on the central nervous system (CNS) and provides significant investments into CNS neuromodulation technologies. These complementary investments and programmatic successes have led to several new and ongoing initiatives for translating these medical devices from concept to clinic. In 2018, the Helping to End Addiction Long-term (HEAL) Initiative published several solicitations for Translational Devices to Treat Pain and supported research on technologies to address opioid use disorder and overdose.

In 2021, the NIH Blueprint for Neuroscience Research launched the MedTech program to advance the development of therapeutic and diagnostic devices for disorders involving the nervous system. These solicitations welcome projects at an early stage of maturity, where applicants have demonstrated proof of concept. This means that the proposed intervention can safely and effectively diagnose or treat the target disorder, e.g., in a small animal model or in vitro model. The program supports projects through several stages of maturity, including building clinical-grade prototypes and performing first-in-human studies of safety and effectiveness. The Blueprint MedTech solicitations remain open to applicants.

While most of NIH ICOs supporting disorders of the nervous system are participating in HEAL and Blueprint MedTech, many have their own ongoing tailored initiatives to better target their research communities. Over 15 ICOs at NIH have funded vagus nerve stimulation research, with NIDDK, NINDS, and NHLBI awarding the most funds (RePorter n.d.). Initiatives of note include the trans-NIH Bioengineering Partnerships with Industry U01 (PAR-24–325), the Trailblazer R21 for early-stage technology development (PA-25–169), and the NINDS-specific Translational Neural Devices R61 / R33 (PAR-25–053). NIH has also historically supported small businesses (through the SBIR and STTR programs) to advance neuromodulation technologies. Potential applicants are always encouraged to talk to NIH program staff to discuss the most appropriate funding opportunities.

SPARC-funded data resources represent another area of continued growth and investment. Four additional NIH initiatives have opted to leverage the SPARC Portal to support their data and knowledge sharing needs. These include the HEAL Restoring Joint Health and Function to Reduce Pain (RE-JOIN) program, the HEAL Program to Reveal and Evaluate Cells-to-gene Information that Specify Intricacies, Origins, and the Nature of Human Pain (PRECISION) program, the NCCIH-funded Topological Atlas and Repository for Acupoint research (TARA), and NCCIH-led NIH Blueprint for Neuroscience Research's Functional Neural Circuits of Interoception. Having recognized the value of the SPARC Portal in supporting other NIH neuroscience initiatives, the NIH Blueprint for Neuroscience Research began co-funding the data resource in 2025 as a standalone grant (U24EB038234) to continue in its role as a convening hub for the field.

Outside of NIH, the Advanced Research Projects Agency for Health (ARPA-H) and the DARPA Biological Technologies Office occasionally solicit ambitious new systems to tackle major challenges preventing the translation of medical technologies, which can be addressed via neuromodulation.

## Discussion

The goal of the NIH Common Fund is to support bold scientific programs that catalyze discovery across all biomedical and behavioral research. The Common Fund creates a space where investigators and multiple NIH ICOs collaborate on innovative research expected to address high priority challenges for the NIH as a whole and make a broad impact in the scientific community. The SPARC program has achieved the overall goals of the Common Fund. SPARC has been transformative by providing much needed investment in the field of neuromodulation in areas that previously challenged the field. Some examples include mapping of nerve connectivity, development of technologies and computational tools to facilitate more rigorous neuromodulation studies, creation of an ecosystem of open specification neuromodulation device components, and development of the SPARC Portal. The SPARC Portal is a platform that includes open sharing and visualization of AI-ready data, functional connectivity maps, comprehensive semantic knowledge bases, and collaboration ready, AI-enabled simulation infrastructure. With the recent NIH emphasis on new approach methodologies, the SPARC Portal is also poised to support further neuromodulation advances through in silico medical device development and the development of human-based digital twins.

The SPARC program has been catalytic in advancing scientific breakthroughs in the field of neuromodulation and in accelerating change through the generation of new data, technologies, and resources that were not available prior to its inception. In fact, the best practices of how SPARC was managed within NIH are continuously shared with other Common Fund programs to facilitate successes in other scientific areas. The goal-driven and synergistic design of the SPARC program allowed scientists with cross-disciplinary and cross-sectoral expertise to focus on time-limited goals to drive the field of neuromodulation forward. Finally, the SPARC program has produced a variety of novel data and resources that are publicly available through the SPARC Portal, providing scientists with cutting-edge information and tools for advancing neuromodulation. Program researchers additionally generated rich data through intricate mapping of different nerves and organ systems, including the anatomy of the functional connectivity of the human vagus nerve. Overall, the products of the SPARC program provide a strong foundation for more efficient clinical studies and effective neuromodulation solutions. The completion of SPARC Phase 2, the SPARC Portal, and other federal investments in bioelectronic medicine offer opportunities for continued innovation and discovery.

The most impactful NIH Common Fund programs are those which lay a foundation and spur further interest in advancing a field of research. Since these programs have a finite lifetime, the long-term success of a program cannot be measured solely from the output of the individual awards. Based on adoption of SPARC outcomes and resources by BRAIN, Blueprint, HEAL, and individual NIH ICO efforts, the authors believe that the SPARC program will be seen as the crystal seed that yielded highly targeted bioelectronic treatments for whole body conditions that were poorly served by small molecules.

Disclaimer: The views and opinions expressed in this manuscript are those of the authors only and do not necessarily represent the views, official policy or position of the U.S. Department of Health and Human Services or any of its affiliated institutions or agencies.

## Methods

### SPARC dataset publication metrics

The number of SPARC datasets shared, dataset downloads, cumulative data quantity, and protocol views were obtained directly from the SPARC Portal on January 12, 2026. These data were obtained using Google Analytics and the Pennsieve platform (which hosts the SPARC Portal). The number of papers published and publication citations were obtained from iCite. In this process a list of SPARC publications were found by searching the iSearch database for PubMed Identifiers (PMIDs) of publications referencing SPARC funded grants. The resulting portfolio was filtered in iCite to remove non-research articles.

### Number of publications and citations

The number of SPARC publications and their citations was calculated by searching the PubMed and iSearch databases for publications citing SPARC awards. The resulting portfolio was filtered to remove non-research articles such as reviews and exported to iCite to calculate the number of citations per year across the portfolio. iCite also reported the relative citation ratio (RCR) for each publication. A weighted RCR greater than the number of publications from that year indicates that a body of literature is highly impactful to the broader research landscape. Data used to generate Fig. 3 was obtained in December of 2025. Data used to generate Table 3 RCR values was obtained from iCite in December of 2026. Publications from 2024 and 2025 were not included in RCR calculations as it is best practice to allow at least a year between publication and citation analysis to allow uptake in the literature.

### Altmetric’s ‘attention score’

Altmetrics provide information on how publications have been received and disseminated online. Altmetric’s ‘Attention Score’ is a weighted count of the attention that a scholarly article has received. It is derived from three main factors: the number of mentions, number of sources that mention the publication, and how often an author mentions the publication. The Altmetrics score for each publication within the SPARC portfolio present in the Altmetrics database, adjusted for its journal of publication and the time at which it was published, were collected through the Altmetrics API using R.

### NIH neuromodulation awards

All NIH awarded extramural grants, Other Transactions Authority awards, and administrative supplements from FY 2014 to FY 2024 with the terms “autonomic nervous system”, “sympathetic nervous system”, “parasympathetic nervous system”, “peripheral nervous system”, “enteric nervous system”, “vagal”, “vagus nerve”, “splanchnic nerve”, “pudendal nerve”, “hypogastric nerve”, “sacral nerve”, “celiac plexus”, “bioelectronic medicine”, or “electroceuticals” in their title or abstract were identified through the NIH Query View Report (QVR) application to construct a general bioelectronic medicine portfolio. Total spending across the entire NIH was calculated using QVR and used to find the fraction of NIH spending on bioelectronic medicine awards. For each award, the individual contribution from separate Institutes, Centers, and Offices (ICOs) was used to calculate what percent of individual ICO spending was spent on bioelectronic medicine awards.

### Cytoscape

The SPARC co-authorship networks were generated using Cytoscape, selecting publications by authors who published at least two publications within the SPARC Phase 1 portfolio. The list of SPARC Phase 1 publications was split into two lists of papers between 2015–2019 and those from 2019–2023. Co-authorship information for these lists was exported from iSearch, which disambiguated all author names. All author disambiguations were manually verified. The co-authorship information was imported to Cytoscape and networks constructed for both sets of publications where nodes represent an author and edges represent the number of co-authorships with any connected authors.

## Data Availability

No datasets were generated or analysed during the current study.

## Abbreviations

AI: Artificial Intelligence
ANS: Autonomic Nervous System
ARPA-H: Advanced Research Projects Agency for Health
BRAIN: Brain Research through Advancing Innovative Neurotechnologies
CFDE: Common Fund Data Ecosystem
CNS: Central Nervous System
DARPA: Defense Advanced Research Projects Agency
FAIR: Findable, Accessible, Interoperable, Reusable
FY: Fiscal Years
HEAL: Helping to End Addiction Long-term
ICOs: Institutes, Centers, and Offices
IDE: Investigational Device Exemption
M: Million
NCATS: National Center for Advancing Translational Sciences
NCCIH: National Center for Complementary and Integrative Health
NHLBI: National Heart, Lung, and Blood Institute
NIA: National Institute on Aging
NIBIB: National Institute of Biomedical Imaging and Bioengineering
NIDDK: National Institute of Diabetes and Digestive and Kidney Diseases
NIH: National Institutes of Health
NINDS: National Institute of Neurological Disorders and Stroke
NINR: National Institute of Nursing Research
OD: Office of the Director
PNS: Peripheral Nervous System
PMID: PubMed Identifier
PRECISION: Program to Reveal and Evaluate Cells-to-gene Information that Specify Intricacies, Origins, and the Nature of Human Pain program
RE-JOIN: Restoring Joint Health and Function to Reduce Pain program
SPARC: Stimulating Peripheral Activity to Relieve Conditions
TARA: Topological Atlas and Repository for Acupoint research
VNS: Vagus Nerve Stimulation
